# An Artificial Reflex Arc That Perceives Afferent Visual and Tactile Information and Controls Efferent Muscular Actions

**DOI:** 10.34133/2022/9851843

**Published:** 2022-02-11

**Authors:** Lin Sun, Yi Du, Haiyang Yu, Huanhuan Wei, Wenlong Xu, Wentao Xu

**Affiliations:** ^1^Institute of Photoelectronic Thin Film Devices and Technology of Nankai University, Tianjin 300350, China; ^2^Key Laboratory of Photoelectronic Thin Film Devices and Technology of Tianjin, Tianjin 300350, China; ^3^Engineering Research Center of Thin Film Photoelectronic Technology, Ministry of Education, Tianjin 300350, China; ^4^National Institute for Advanced Materials, Tianjin 300350, China

## Abstract

Neural perception and action-inspired electronics is becoming important for interactive human-machine interfaces and intelligent robots. A system that implements neuromorphic environmental information coding, synaptic signal processing, and motion control is desired. We report a neuroinspired artificial reflex arc that possesses visual and somatosensory dual afferent nerve paths and an efferent nerve path to control artificial muscles. A self-powered photoelectric synapse between the afferent and efferent nerves was used as the key information processor. The artificial reflex arc successfully responds to external visual and tactile information and controls the actions of artificial muscle in response to these external stimuli and thus emulates reflex activities through a full reflex arc. The visual and somatosensory information is encoded as impulse spikes, the frequency of which exhibited a sublinear dependence on the obstacle proximity or pressure stimuli. The artificial reflex arc suggests a promising strategy toward developing soft neurorobotic systems and prostheses.

## 1. Introduction

Artificial sensory and motor nervous systems are applicable to replace damaged nerves of disabled persons, to enhance human sensory and motor functions, and to provide solutions to compatible human-machine interface and neurorobots [1–6]. So far, partial functions have been realized for environmental signal perception [7–9], neuron-like signal transmission and processing [7–10], and action control [10, 11].

To perceive informative stimuli in real environments, an artificial peripheral system must be able to detect inputs, encode them into spiking signals that mimic action potentials, transmit the signals, interface with synaptic processing units, and then send orders to induce the motor system to react. Previous reports on artificial neural systems have used a microcomputer [12] or ring oscillators [7, 8, 13–15] to encode tactile sensory information and ion-exchange polymer metal composite (IPMC) for actuation [10, 16–18]. However, synaptic connections with afferent sensory inputs and efferent motor control that combine to form a full reflex arc have been less reported. Moreover, to further make the system suitable for biointegrated applications, self-powered synaptic processing units are attractive because they reduce energy consumption of the system [19]. Multisensory information perception can establish accurate descriptions of the environment, so multimodal fusion can improve the accuracy of recognition and classification and environmental-response interactions [14, 20–23]. Therefore, to form a complete sensory-to-motor pathway with multiple sensory-modality coding, a full reflex arc that replicates versatile peripheral functions is desired.

Here, we present an artificial reflex arc that senses visual and tactile information, processes these signals by using a self-powered optoelectronic perovskite (PSK) artificial synapse, and controls artificial muscular actions in response to environmental stimuli. Our optoelectronic perovskite synaptic devices emulate important synaptic functions. Inspired by the sensory and motor pathways in biological nervous systems, the artificial reflex arc consists of visual and somatosensory dual afferent neural pathways and an efferent neural pathway, by integrating a self-powered photoelectric synapse with front-end sensors and backend artificial muscle. Visual and somatosensory information was encoded into impulse spikes at similar frequencies of action potentials, with the spiking frequency exhibiting a sublinear dependence on the obstacle proximity or on pressure stimuli. The reflex arc controls the contraction of artificial muscle fibers in response to stimuli from either visual or somatosensory pathways. The artificial reflex arc may improve the interaction between humans or neurorobots with external environments. The design provides a new pathway to future neurorobots, neural interfaces, and artificial prosthesis.

## 2. Results

### 2.1. Configuration of Neuroinspired Artificial Reflex Arc

**Figure 1:**
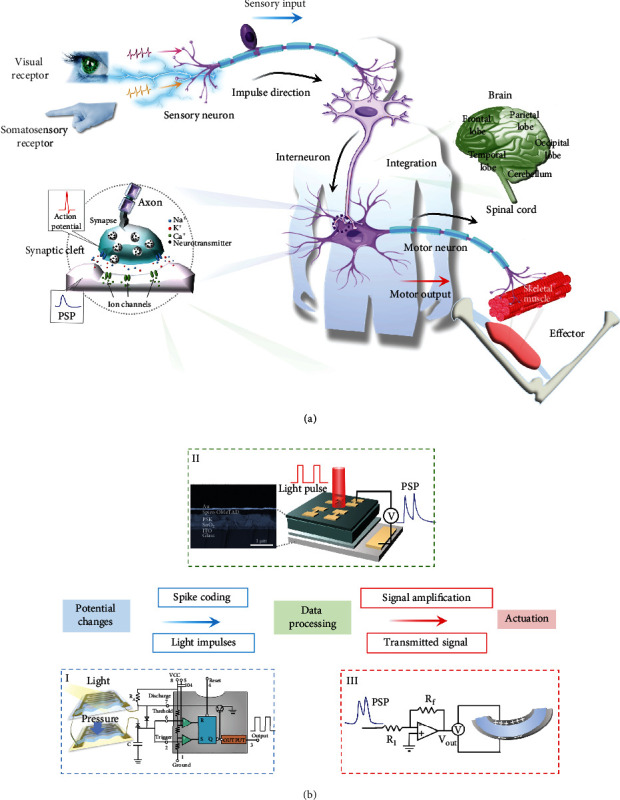
Sensory and motor pathway in the biological nervous system and neuromorphic signal transmission. (a) Sensory and motor pathways; the part highlighted is the schematic illustration of biology synapse structure. (b) (i) Schematic of design of in artificial sensorimotor nerve, (ii) operation mode (right) and cross-sectional scanning electron micrograph of structure (left), and (iii) signal transmission. Information from visual and somatosensory stimuli is transformed to a neuromorphic signal, which stimulates peripheral nerves that actuate an artificial muscle response.

In a biological nervous system, sensory receptors detect information about the surroundings, and then, the nervous system processes the information and decides how to respond (Figure 1(a)). The central and peripheral nervous systems have important functions in these processes. Neurons are the basic functional units of the nervous system; they propagate signals by generating depolarizations of the neurilemma, i.e., action potentials, which are voltage spikes that propagate along axons. Stimulus information is encoded in patterns of action potentials and transmitted to and within the brain. Inspired by the sensory and motor pathway in the biological nervous system, we demonstrate an artificial sensorimotor nerve that includes both a visual pathway and a somatosensory neural pathway by integrating a self-powered photoelectric synapse, sensors (light-dependent or pressure-dependent resistor) and a polymer actuator. We mimic the visual and somatosensory neuromorphic signal transmission by a multivibrator circuit and an amplifier circuit (Figure 1(b)).

A synapse is a neural structure that passes an electrical or chemical signal to another neuron or to the target effector cell. The synapse is composed of the axon ends of the presynaptic neuron, the dendrites or cell bodies of the postsynaptic neuron, and the synaptic cleft between them (Figure 1(a)). When an appropriate action potential reaches the presynaptic neuron, voltage-gated calcium ion channels open, and a large amount of Ca^2+^ floods into the membrane to trigger the release of neurotransmitters into the synaptic cleft. Each type of neurotransmitter binds to a specific postsynaptic receptor protein and induces the opening of the transmitter-gated ion channel. Depolarization of the postsynaptic cell by permeable ions (e.g., Na^+^ and K^+^) is called excitatory postsynaptic potential (EPSP), whereas hyperpolarization of the postsynaptic cell by permeable ions (e.g., Cl^−^) is called inhibitory postsynaptic potential (IPSP). The basic structure of an optoelectronic synaptic device is very similar to that of a biological synapse: in the optoelectronic perovskite synaptic device, the top electrode corresponds to the presynaptic membrane, the bottom electrode corresponds to the postsynaptic membrane, pulses of light can be regarded as action potentials that act on the presynaptic neuron, and the charge carriers in the active material between the two electrodes are equivalent to neurotransmitters (Figure 1(b), ii). Similarly, stimulation by light can cause an increase or decrease in the conductance of the active material, resulting in excitatory postsynaptic potential (EPSP), excitatory postsynaptic current (EPSC), inhibitory postsynaptic potential (IPSP), or inhibitory postsynaptic current (IPSC) [24]. The perovskite synaptic device (Figure 1(b), ii) consists of ITO, electron transport layer SnO_2_, PSK, hole transport layer Spiro-OMeTAD, and Au layers. Current-voltage *I*-*V* measurements (Figure S2a) of the perovskite device showed a sharp increase in *I* after *V* exceeded 0.75 V; at *V* < 0.75 V, *I* was 0 in darkness, and about -2 *μ*A under illumination. The external quantum efficiency (EQE) was ~80% at wavelengths (450 ≤ *λ* ≤ 800 nm) (Figure S2b). The hybrid structure of PSK effectively absorbs light in the spectral range from ultraviolet (UV) to near-infrared (Figure S2c, d).

### 2.2. Self-Powered Synaptic Characteristics of Optoelectronic Perovskite Device

**Figure 2:**
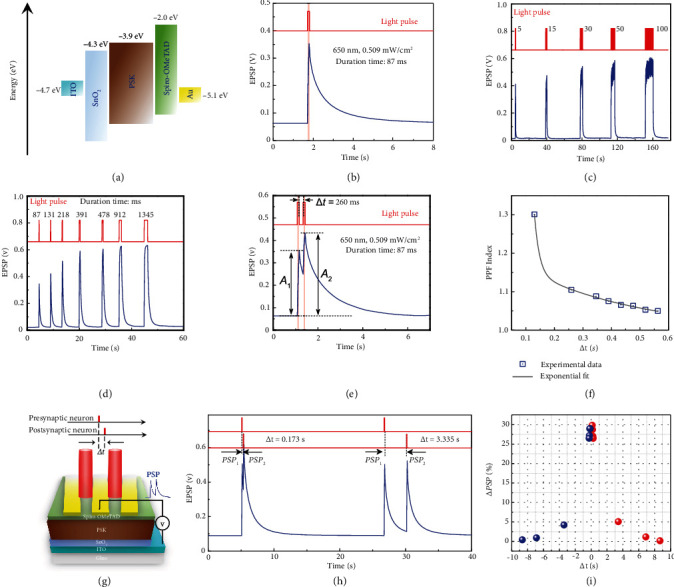
Self-powered synaptic characteristics of optoelectronic perovskite device. (a) Energy diagram of perovskite synaptic device. (b) Transient EPSP of perovskite synaptic device in response to one optical spike. (c) EPSP vs. number of optical spikes. (d) EPSP vs. duration of optical spikes. (e) Change in EPSP in response to a pair of presynaptic optical spikes. *A*_1_ and *A*_2_ represent the change in PSP at the first and second spike, respectively. (f) Paired-pulse facilitation (PPF) index vs. time interval between pairs of optical spikes. Emulation of symmetric spike-timing-dependent plasticity (STDP). (g) Schematic showing two connected perovskite synapses for emulation of STDP. (h) The EPSP values at different Δ*t*. (i) Variation of connection strength between the presynaptic and postsynaptic devices as a function of Δ*t*.

Compared with the reported electrically driven neuromorphic devices, synaptic devices that respond to light stimuli may be advantageous in having wider bandwidth, low crosstalk, and better scalability than conventional electrically-stimulated devices and may contribute to increasing in the computational speed of neural networks. Moreover, to further make the system suitable for biointegrated applications, self-powered synaptic processing units are attractive because they reduce the need to use batteries [19]. In the hybrid-structured synaptic device, optical spike generates excess electrons and holes in PSK (Figure 2(a)). The heterojunction separates the photo-generated electrons from the holes. Built-in electric fields occur between Spiro-OMeTAD and SnO_2_; the fields drive photo-generated electrons toward the ITO and holes toward the Au. This photovoltaic effect yields a corresponding EPSP or EPSC with the external light stimulation; i.e., a photoelectric synapse that has the structure ITO/SnO_2_/PSK/Spiro-OMeTAD/Au enables it to work without an electrical power supply.

Defects at the interface between layers may trap photo-generated electrons in the PSK. The subsequent trapped electrons lead to a decay of the EPSP or EPSC. The change of postsynaptic potential (PSP) increases under light stimuli (Figure 2(b)); this corresponding change indicates the excitatory postsynaptic potential and its potentiation of the synaptic strength. The subsequent PSP returns to its initial value when the light stimuli are removed; this decay emulates the typical short-term plasticity (STP) that biological synapses show. The photoelectric synaptic plasticity was also shown by tuning the light-stimulation parameters, including the number of light pulses (Figure 2(c)), width of light pulse (Figure 2(d)), and intensity of light (Figure S7).

Paired-pulse facilitation (PPF) is an important manifestation of short-range plasticity. It refers to an increase in postsynaptic response after two consecutive synaptic stimulations (Figure 2(e)). In a biological nervous system, PPF contributes to recognition and decoding of time-resolved information, such as visual and auditory signals. The biological synaptic facilitation gain increases as the time interval Δ*t* between stimuli decreases (Figure 2(f)). PSP gain can be measured using PPF index (*A*_2_/*A*_1_, where *A*_2_ and *A*_1_ are, respectively, the amplitudes of the second and first pulses); its dependence on Δ*t* follows a double exponential function:

(1) $$\begin{array}{c} \text{PPF}\;\;\text{index}=C_{1}\;\;\exp\;\;\left(-\frac{\Delta t}{\tau_{1}}\right)+C_{2}\;\;\exp\left(-\frac{\Delta t}{\tau_{2}}\right), \end{array}$$

in which *C*_1_ and *C*_2_ are initial facilitation magnitudes, *τ*_1_ is the time constant of rapid decay, and *τ*_2_ is the decay constant of slow decay. At Δ*t* smaller than the EPSP decay time, *τ*_1_ = 0.017 s and *τ*_2_ = 0.4 s. *τ*_2_ is about one order of magnitude larger than *τ*_1_; this difference is consistent with the characteristics of the decay of the PPF index in a biological synapse [25, 26].

The Hebbian model of learning requires both activity-dependent synaptic plasticity and a mechanism that induces competition among synapses. Spike-timing-dependent plasticity (STDP) is a form of synaptic plasticity that depends on the relative timing of pre- and postsynaptic action potentials (Δ*t* = *t*_pre_ − *t*_post_) [26–28]. STDP can be either symmetric or asymmetric. To emulate the STDP function, two PSK synaptic devices were configured (Figure 2(g)) to share an electrode. The one side is considered a presynaptic neuron, and the other side is considered a postsynaptic neuron. A 650 nm light pulse was beamed separately at each device; Δ*t* was controlled by varying the time interval between the two optical spikes, from -8.6 s (presynaptic neuron stimulated first) to +8.6 s (postsynaptic neuron stimulated first). The connection strength between neurons was defined as ΔPSP = (PSP_2_ − PSP_1_)/PSP_1_ (Figure 2(h)). Δ*t* affected the connection strength between the two devices (Figure 2(i)). A symmetric STDP behavior was observed in which the *Δ*PSP was highest when ∣Δ*t*∣ was small and weakened as ∣Δ*t*∣ was increased. This simple symmetric STDP characteristic is a basic requirement for emulation of brain functions. In addition, our synaptic device also exhibited the capability of cyclic endurance and reliability (Figure S9).

### 2.3. Encoding Stimulus Information in Artificial Sensory Nervous System

Sensory neurons regulate their activities by triggering sequences of action potentials in various time patterns when activated by sensory stimuli. Encoding multimodal sensory information about their surroundings can improve the interaction of humans or robots with objects. Vision is an important way for animals to perceive external information.

The somatosensory system responds to changes at the surface or inside the body. Light sensors and pressure sensors send stimulus signals along sensory paths to the place where they may be processed by certain sensory neurons and then relayed to the brain for further processing. In a “neuromorphic” tactile sensory system, encoding stimulus information is similar to that used by human nerves in response to tactile stimuli, so the artificial neuromorphic device outputs can replace them and communicate with other motor neurons or residual nerve fibers of amputees [7]. In this scenario, with two levels (visual and somatosensory), objects can be better recognized.

**Figure 3:**
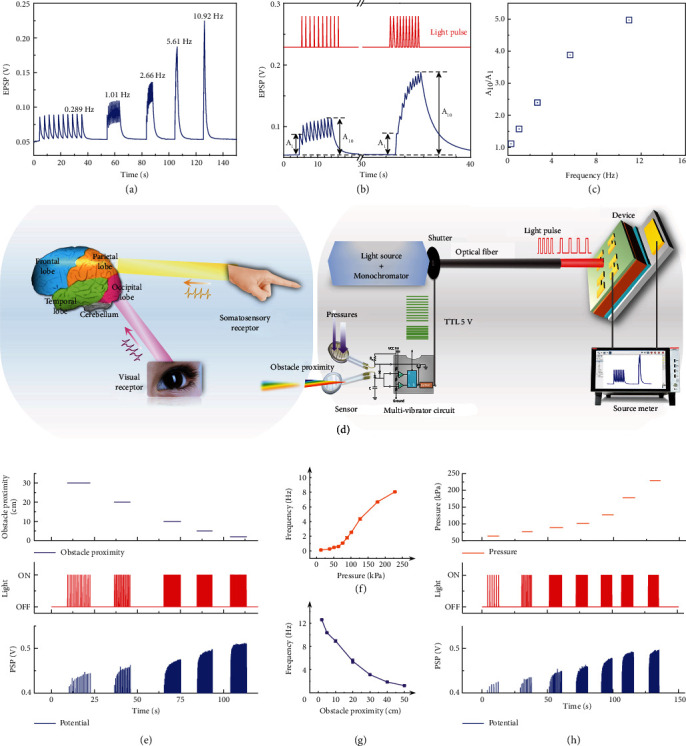
Encoding visual and somatosensory stimulus information in artificial sensory nerve. (a) Spike frequency-dependent EPSP amplitude triggered by a train of 10 optical spikes. (b) *A*_1_ and *A*_10_ represent the amplitudes of the first and tenth ∆PSPs, respectively. (c) The EPSP gain (*A*_10_/*A*_1_) plotted vs. spike frequency of light. (d) Schematics of visual and somatosensory stimulus information in the human brain and neural coding of visual and somatosensory information. Schematic showing the visual and somatosensory information to the sensor/multivibrator circuit, which converted them into streams of electrical pulses and then drives the shutter into streams of light pulses to stimulate synaptic device. Frequency output as a function of the pressure (f) and obstacle proximity (g) applied to the sensors. Correlation among EPSP, light pulse output, and pressure (e) and obstacle proximity (h).

In most sensory systems, the rate-coding model of neuronal firing states that the frequency or rate of action potentials increases and generally nonlinearly as stimulus intensity increases [25–30]. From the spike frequency-dependent EPSP amplitude (Figures 3(a)–3(c)), our photoelectric synapse demonstrates a similar relationship between a stimulus and EPSP gain. Photoelectric synaptic plasticity was tunable by light-stimulation parameters, including the spike frequency (Figures 3(a)–3(c)), the number of light pulses (Figure 2(c)), width of light pulse (Figure 2(d)), and intensity of light (Figure S7). Compared with light intensity stimulation, the frequency coding of optical spikes is a more energy-efficient, and frequency-encoded information can be more robust to voltage degradation and parasitic resistances than amplitude-encoded information [7]. Therefore, this transduction in the form of frequency coding is inherently power-efficient and robust to noise [8]. Here, we assume that the stimulus information is contained in the firing rate of neuron. Our artificial sensory neurons can be excited at two levels (visual and somatosensory) by encoding in electrical signals that mimic action potentials (Figures 3(f) and 3(g)). A myoelectric prosthesis inspired by this artificial sensory neuron may utilize the residual neuromuscular system of the human body to control the functions of an electric prosthetic limb [31–34].

Here, an artificial sensory nervous system is designed by integrating the sensor/multivibrator circuit and an optoelectronic synaptic device (Figure 3(d)). The sensor/multivibrator circuit serves as a sensory receptor to transform visual and somatosensory stimuli into electric pulses, and the optoelectronic synaptic device serves as a synapse to further process the information. The sensory nervous system exhibits excellent multisensory stimulation, whereas the self-powered optoelectronic perovskite synapses realize fundamental synaptic functions without additional power consumption. In addition, the front-end sensor of our system uses passive components, which is beneficial to further reduce power consumption. Passive components and self-powered synaptic processing units are attractive, in combination with parallel information processing with spikes, leading to the scalability of our artificial system; i.e., the number of receptors and artificial neurons increase without causing an appreciable increase in the latency and power consumption. The sensory nervous system can detect visual and somatosensory information stimuli that are encoded as temporal information, such as pressure (Figure 3(e)) and obstacle proximity (Figure 3(h)). In the artificial sensory nerve, the frequency of spikes increased as pressure increased (Figure 3(f)) or decreased as obstacle distance increased (Figure 3(g)). These responses could be useful in neuromorphic signal transmission that imparts interactive experience hierarchically in neurorobotics or detects a potential collision and triggers a timely escape response in the field of autonomous vehicle safety. Due to the limitation of mechanical shutter, the operating frequency range of the system is less than 20 Hz. A diode light source or pulsed optical fiber light source could be used in future design to input higher frequency optical spikes.

### 2.4. Optoelectronic Sensorimotor Nervous System with Optical Stimulation of Motor Neurons

**Figure 4:**
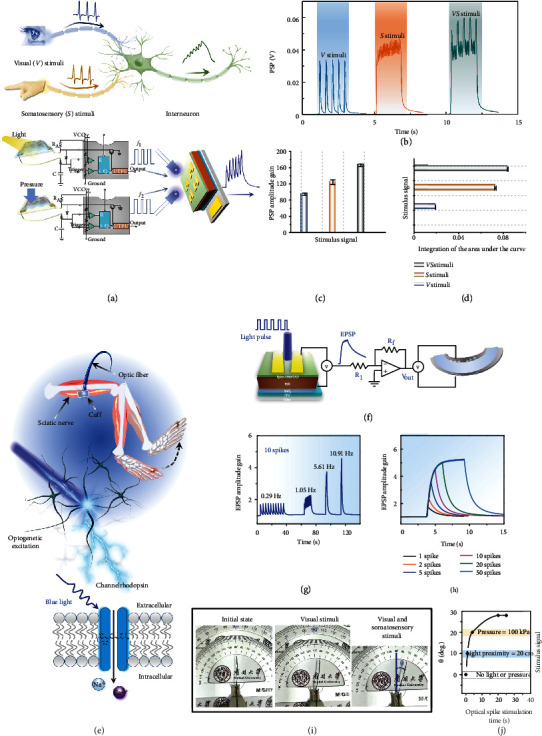
Optical stimulation of motor neurons. (a) Visual (*V*) and somatosensory (*S*) afferents and the integrative function of the interneurons. (b) Characterization of a dual-channel artificial reflex arc with visual and somatosensory stimuli. PSP amplitude gain (c) and area of the postsynaptic integration signal (d) under visual and somatosensory stimuli. The integration of signals from two stimuli by photoelectric synapse improves the discrimination among the information. Actuators can respond by a larger margin and reach the operating threshold faster. (e) Optical excitation for optogenetic control of the peripheral nervous system and schematics of optogenetic excitation. (f) Mimicking muscle function using optical stimulation of motor neurons. Schematics and overall configuration of connection of optoelectronic perovskite synaptic device and a motor unit. (g) Spike frequency-dependent EPSP amplitude and (h) spike number-dependent EPSP amplitude of optoelectronic sensorimotor nervetronics. (i) Photograph of angular displacement of a polymer actuator (artificial muscle effector) according to the stimuli-dependent EPSP amplitude. (j) Statistical curve of polymer actuator deflection. Specifically, when the light proximity distance is 20 cm, the optical spike frequency is ~5.5 Hz; while the pressure is 100 kPa, the optical spike frequency is about 3 Hz.

The nervous system obtains information from the external environment and transmits signals to respond to corresponding motion feedback. The vital interneuron interprets and integrates the input information from the receptors and sends an efferent impulse to an efferent neuron, such as a motor neuron (Figure 4(a)). Such processing and integration of multimodal information about the surroundings can improve the interaction of humans or robots with objects. The photoelectric synapse can combine signals from multiple stimuli (Figure 4(b)). From PSP amplitude gain (Figure 4(c)) and area of the postsynaptic integration signal (Figure 4(d)) in response to visual and somatosensory stimuli, the integration of signals from two stimuli by photoelectric synapse improves the discrimination among the information, so the actuator's margin of response can increase and decrease the time taken to reach the operating threshold.

Sensorimotor nervetronics is the integration of synaptic electronics and artificial sensory-motor systems to produce a motion response similar to a biological body (reflex arc or motor neuron-related muscle contraction) [10]. This will promote humanoid robots, neuroprosthetics, and biocompatible exoskeletons.

Optogenetics provides millisecond-level optical control of the neural activity of specific cell types during animal behavior. In some cases, at single-cell resolution, its remarkable accuracy allows manipulation of the activities of conscious animals and investigation of causal connections in neural circuits [35–40]. Optogenetics approach is promising to restore the motor function of damaged neuromuscular systems by optical stimulation of motor neurons that are genetically modified to be photosensitive (Figure 4(e)) [35–40]. Stimulating muscle fibers with light can achieve more precise control than neuromuscular electrical stimulation. In theory, optogenetics can treat a variety of movement disorders in the future [35–40]. The operation voltages of artificial sensory synapses can be reduced by adopting a configuration that uses a self-powered optoelectronic perovskite synaptic device that detects the optical signals, then generates PSP (Figure 4(f)). A self-powered optoelectronic perovskite synaptic device was used as an artificial light receptor, and the voltage that it output under light stimulation was used as a postsynaptic potential. As the number and frequency of optical pulses increased, the EPSPs were amplified and thus induced accumulation of photogenerated electron-hole pairs in the channel (Figures 4(g) and 4(h)). These device behaviors can be used to mimic the activation response of biological muscles [10]. Motor neurons send signals to muscle fibers via a neuromuscular junction to cause muscle contraction [10]. The intensity of a muscle contraction increases as the frequency of action potentials increases [35–40]. Therefore, we can regulate the motor response by the frequency at which the stimulus is converted into an action potential. The self-powered photoelectric synapse can be subsequently used to interface with an artificial muscle effector to form a complete visual and somatosensory dual neural pathways reflex arc.

Artificial motor neurons composed of an artificial optoelectronic perovskite synaptic device and artificial muscle fiber (a polymer actuator) can mimic this contraction response of biological muscles by receiving presynaptic action potentials from a light-sensory optoelectronic perovskite device, then transferring postsynaptic signals to an artificial muscle fiber (Figure S5). Postsynaptic output voltage and actuation of artificial muscle effector were controlled by the visual and somatosensory dual neural pathways stimuli and showed contraction responses that were similar to those of real skeletal muscle (Figure 4(i)). Specifically, deflection angle *θ* with no stimuli at 0 deg. was increased to ~20 deg. with *VS* stimuli for a short time (Figure 4(i)). The polymer actuator operated stably after *VS* stimuli with spike time beyond ~23 s and deflection gradually reached the maximum angle (Figure 4(j)). Our artificial sensorimotor system reported here combines multimodal sensing information, processing and motor driving. It is a good example of autonomous driving. The recently reported automatic escape system also uses neuromorphic devices (floating-gate memory transistor [41] and two-terminal threshold switching memristor [42]). Furthermore, scientists have combined brain-inspired neural computation principles and scalable deep learning architectures to design compact neural controllers for task-specific compartments of a full-stack autonomous vehicle control system [43].

## 3. Discussion

We demonstrated a neuroinspired artificial reflex arc that integrates sensory nerves with visual and somatosensory dual neural pathways and a motor nerve that sends orders to stimuli-responsive artificial muscle. The system integrates a self-powered photoelectric synapse with front-end light and pressure sensors and backend polymer actuators. Compared with the previous artificial nervous system (Table S1, Supporting Information), we firstly propose a full reflex arc with multiple sensory-modality coding. The design is a versatile and extensible architecture. In this system, a self-powered optoelectronic synapse was adopted to percept optical stimulation. The artificial reflex arc encodes visual and somatosensory afferent information into impulsive spikes at different frequencies similar to the process in biological sensory nerves, processes the information using the photoelectric synapse unit, and then sends efferent orders to control the actions of artificial muscle. The output spiking frequency exhibited a sublinear dependence on the obstacle proximity or pressure stimuli. Encoding multimodal sensory information of the surroundings and the stimuli-responsive artificial muscular functions can improve the interaction of humans or robots with external environments. Our artificial reflex arc suggests a promising strategy toward developing bioinspired electronics, neurorobotics systems, and artificial prosthesis.

## 4. Materials and Methods

### 4.1. Materials

Lead iodide (PbI_2_), bis(trifluoromethylsulfonyl)-imide lithium salt (Li-TFSI), chlorobenzene, 4-tert-butylpyridine, *N*,*N-*dimethylformamide (DMF), dimethylsulfoxide (DMSO), and isopropyl alcohol (IPA) were purchased from Sigma-Aldrich. Formamidinium iodide (FAI), methylammonium bromide (MABr), and methylammonium chloride (MACl), 2,2′,7,7′-tetrakis-(*N*,*N-*di-4-methoxyphenylamine)-9,9′-spirobifluorene (Spiro-OMeTAD) were purchased from Xi'an Polymer Light Technology Corp. 2-Methoxyethanol was purchased from Aladdin. Tin (IV) oxide colloid precursor was purchased from Alfa Aesar.

### 4.2. Fabrication of Optoelectronic Perovskite Synaptic Device

ITO glass was cleaned using detergent water, deionized water, acetone, and then isopropanol for 15 min each sequentially in ultrasonic washer. An SnO_2_ (diluted by water to 3.75 wt%) layer was deposited on ITO substrates by spin-coating at a speed of 4,000 rpm for 30 s, then annealed at 150°C for 30 min. The ITO substrates were treated with UV for 10 min; then 1.5 M PbI_2_ in mixed solution of polar solvents (DMF : DMSO = 9 : 1, *v*/*v*) was spin-coated on the SnO_2_ layer at 1,500 rpm for 30 s. The PbI_2_ film was annealed at 70°C for 1 min. The organic salts (FAI : MABr : MACl = 90 mg : 6 mg : 9 mg in 1 mL IPA) were deposited on the PbI_2_ layer at 1,600 rpm for 30 s; then, the films were transferred in ambient air and annealed at 150°C for 20 min. Then, the films were transferred into a N_2_ glove box and spin-coated with Spiro-OMeTAD at 3,000 rpm for 30 s. The hole-transport material was deposited by preparing 72.3 mg Spiro-OMeTAD in chlorobenzene and mixing with 35 *μ*L Li-TFSI (260 mg/mL in acetonitrile) and 30 *μ*L 4-tert-butylpyridine. An Au electrode (80 nm) was deposited using thermal evaporation.

### 4.3. Fabrication of PVDF-HFP/EMIMBF_4_ Electrolyte Layer

An electrolyte layer was fabricated by dissolving 1 g poly(vinylidene fluoride-co-hexafluoropropylene) (PVDF-HFP) (Sigma-Aldrich) and 2 g 1-Ethyl-3-methylimidazolium tetrafluoroborate (EMIBF_4_) (Aladdin) in 2 mL N,N-Dimethylformamide (DMF) (Meryer (Shanghai) Chemical Technology Co., Ltd.) at 60°C for 24 h. A glass mold (70 mm x 35 mm) was used to fabricate polymer electrolyte by solution casting.

### 4.4. Construction of CNT/Electrolyte/CNT-Based Electrochemical Actuator

To achieve the polymer actuators, the electrolyte was sandwiched between two CNT electrode-layers, then pressed at 70°C for 2 min. The final actuator was aged under reduced pressure at room temperature for 1 d, then cut into 20 mm × 2 mm strips for further measurement.

### 4.5. Construction of Synaptic Device-Amplifier Circuit-Polymer Actuator System

To operate the polymer actuator, we used an operational amplifier to output the desired voltage. The bottom electrode of synaptic device was connected to the amplifier circuit to convert currents to output voltages, such that the polymer actuator can be operated. The amplifier circuit amplifies the input voltage by 10 times to reach the working voltage of the actuator (~3 V).

### 4.6. Material Characterization and Device Measurements

SEM was performed using a QUANTA FEG 450 field-emission microscope. EQE was measured using an Enli Tech (Taiwan) EQE measurement system. All electrical measurements were characterized using a Keithley 4200A semiconductor parameter analyzer in a N_2_-filled glove box with moisture and oxygen content both <0.1 ppm. Monochromatic light was obtained from a 150 W xenon lamp with corresponding monochromators. Light illumination intensities were calibrated using a standard silicon optical power meter.

## Data Availability

The data used to support the findings of this study are available from the corresponding author upon request.

## Supplementary Materials

Supplementary Materials Figure S1: fabrication of perovskite synaptic device. Figure S2: (a) *I-V* measurements of hybrid perovskite device. (b) EQE measurement of the hybrid perovskite device. (c) UV-vis absorbance spectra of perovskite. (d) Corresponding K-M transformation of the UV-vis absorption spectra of perovskite to calculate the band gap. Figure S3: sensors and multivibrator circuit. Figure S4: resistances of (a) light sensor vs. obstacle proximity and (b) pressure sensor vs. pressure. Figure S5: construction of synaptic device-amplifier circuit-polymer actuator system. Figure S6: self-powered synaptic characteristics of optoelectronic perovskite device. (a) Transient EPSC of perovskite synaptic device in response to one optical spike. (b) EPSC vs. spike duration. (c) Change in EPSC in response to a pair of presynaptic optical pulses. *A*1 and *A*2 represent change in PSC at first and second spike, respectively. (d) Paired-pulse facilitation (PPF) index vs. interval between pairs of excitatory stimuli. (e) EPSC vs. number of optical spikes. (f) Spike frequency-dependent EPSC amplitude triggered by a train of 10 light spikes. Figure S7: the synaptic characteristics of optoelectronic perovskite device measured at different incident light intensities under illumination using light of wavelength 650 nm. (a) PSC and (c) PSP values vs. intensity optical spikes. Postsynaptic current (*b*)/potential (*d*) vs. light irradiation power. Figure S8: the synaptic characteristics of optoelectronic perovskite device vs. incident light wavelengths at 0.5 mW/cm^2^ incident light intensities. The (a) PSC and (c) PSP during 0.5 mW/cm^2^ intensity optical spikes with wavelengths of 450 nm, 520 nm, or 650 nm. (b) PSC and (d) PSP values vs. duration at various incident light wavelengths. Figure S9: (a) EPSP vs. number of optical spikes with *f* = 0.3 Hz. (b) Variation of the off- and on-PSP from the initial to 300 of programming/erasing cycles with an interval of 10 cycles in the endurance performance test. The device is programmed by the on-light pulse (*f* = 0.3 Hz), erased by the off-light pulse, exhibiting the capability of cyclic endurance. (c) EPSP vs. number of optical spikes with *f* = 11.5 Hz [44–48].
