# Supplementary material for: An Artificial Reflex Arc That Perceives Afferent Visual and Tactile Information and Controls Efferent Muscular Actions
Source: Research (Wash D C). 2022 Feb 11;2022:9851843. doi: 10.34133/2022/9851843 (PMC8858381; doi:10.34133/2022/9851843)
Supplement: Supplementary Materials — Figure S1: fabrication of perovskite synaptic device. Figure S2: (a) I-V measurements of hybrid perovskite device. (b) EQE measurement of the hybrid perovskite device. (c) UV-vis absorbance spectra of perovskite. (d) Corresponding K-M transformation of the UV-vis absorption spectra of perovskite to calculate the band gap. Figure S3: sensors and multivibrator circuit. Figure S4: resistances of (a) light sensor vs. obstacle proximity and (b) pressure sensor vs. pressure. Figure S5: construction of synaptic device-amplifier circuit-polymer actuator system. Figure S6: self-powered synaptic characteristics of optoelectronic perovskite device. (a) Transient EPSC of perovskite synaptic device in response to one optical spike. (b) EPSC vs. spike duration. (c) Change in EPSC in response to a pair of presynaptic optical pulses. A1 and A2 represent change in PSC at first and second spike, respectively. (d) Paired-pulse facilitation (PPF) index vs. interval between pairs of excitatory stimuli. (e) EPSC vs. number of optical spikes. (f) Spike frequency-dependent EPSC amplitude triggered by a train of 10 light spikes. Figure S7: the synaptic characteristics of optoelectronic perovskite device measured at different incident light intensities under illumination using light of wavelength 650 nm. (a) PSC and (c) PSP values vs. intensity optical spikes. Postsynaptic current (b)/potential (d) vs. light irradiation power. Figure S8: the synaptic characteristics of optoelectronic perovskite device vs. incident light wavelengths at 0.5 mW/cm2 incident light intensities. The (a) PSC and (c) PSP during 0.5 mW/cm2 intensity optical spikes with wavelengths of 450 nm, 520 nm, or 650 nm. (b) PSC and (d) PSP values vs. duration at various incident light wavelengths. Figure S9: (a) EPSP vs. number of optical spikes with f = 0.3 Hz. (b) Variation of the off- and on-PSP from the initial to 300 of programming/erasing cycles with an interval of 10 cycles in the endurance performance test. The device [file 9851843.f1.docx]

# Supplementary Materials for

**An artificial reflex arc that perceives afferent visual and tactile information and controls efferent muscular actions**

Lin Sun, Yi Du, Haiyang Yu, Huanhuan Wei, Wenlong Xu, Wentao Xu*

*Corresponding author. Email: wentao@nankai.edu.cn, bnuch@hotmail.com

**I. Fabrication of optoelectronic perovskite synaptic device and measurement. (Fig. S1, S2)**


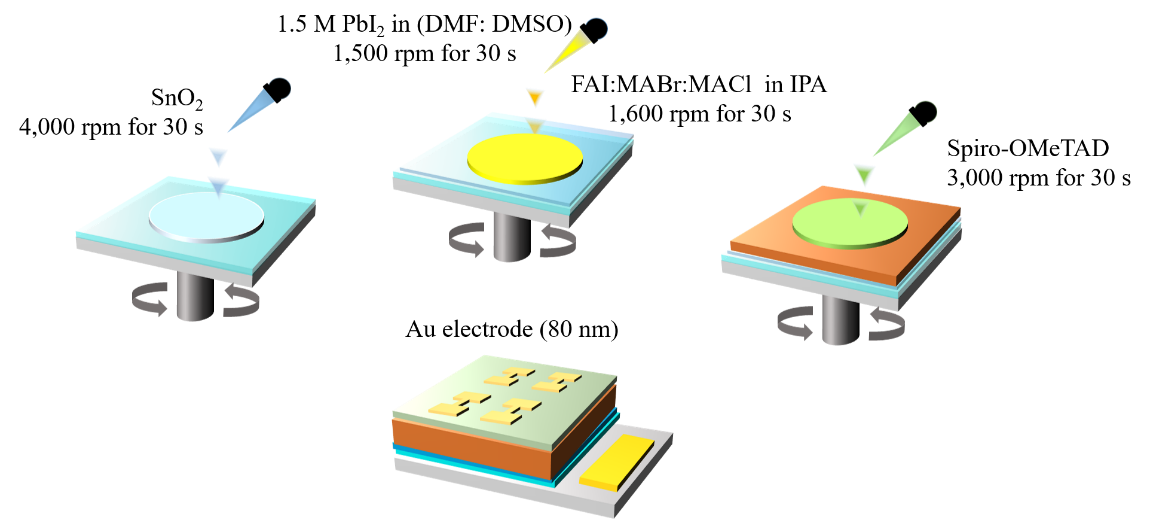


Fig. S1. Fabrication of perovskite synaptic device.


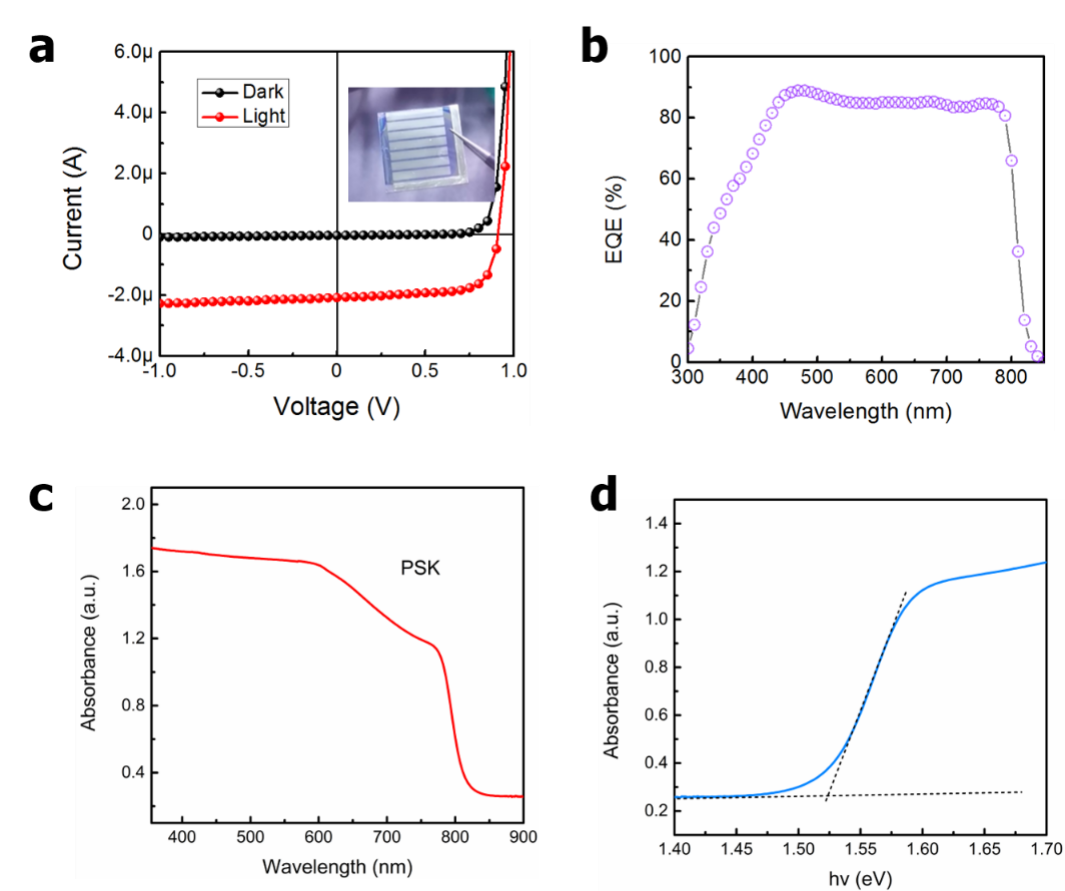


Fig. S2. (a) *I-V* measurements of hybrid perovskite device. (b) EQE measurement of the hybrid perovskite device. (c) UV-vis absorbance spectra of perovskite. (d) Corresponding K-M transformation of the UV-vis absorption spectra of perovskite to calculate the band gap.

**II. Mimic the visual and somatosensory information via sensor and multi-vibrator circuit. (Fig. S3, S4)**

Encoding stimulus information by pulse frequency in artificial sensory nervous system.

Here, we mimic the visual and somatosensory neural communication by integrating our artificial synapses with a sensor (light-dependent or pressure-dependent resistor) by an improving multi-vibrator circuit (Fig. S3). Commercially available sensors were used to ensure operational stability of the system. Our pressure sensor belongs to DF9-series (Leanstar-tech). The initial resistance of pressure sensor exceeds 10 MΩ. A commercial photosensitive resistor was used (T5506) with a dark resistance of 0.2 MΩ.


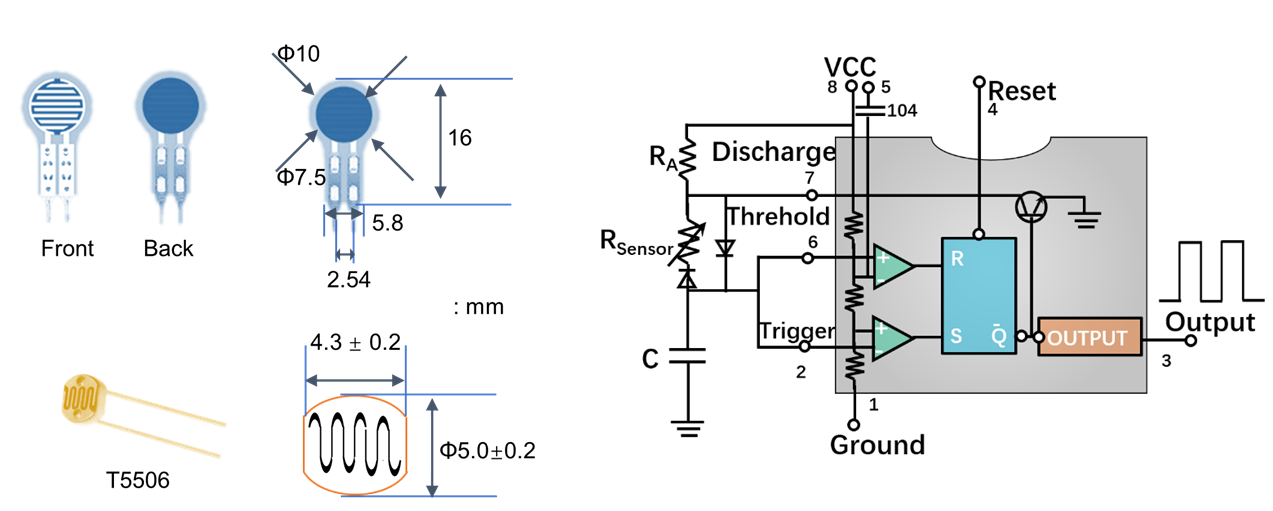


Fig. S3. Sensors and multi-vibrator circuit.


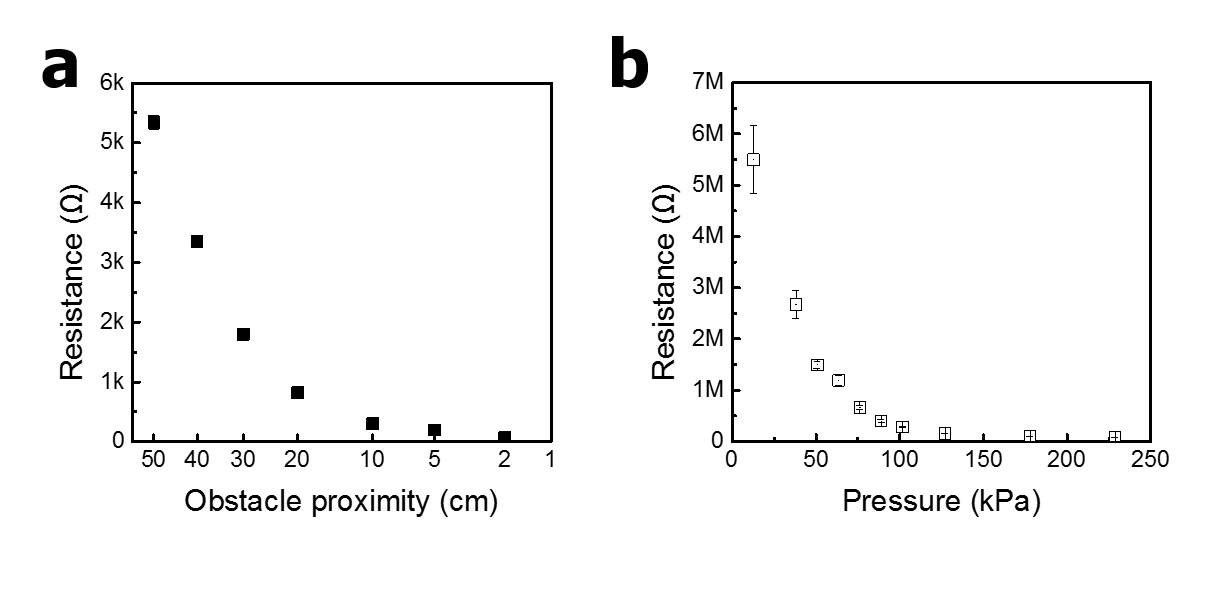


Fig. S4. Resistances of (a) light sensor vs. obstacle proximity, and (b) pressure sensor vs. pressure.

**III. Construction of synaptic device-amplifier circuit-polymer actuator system. (Fig. S5)**


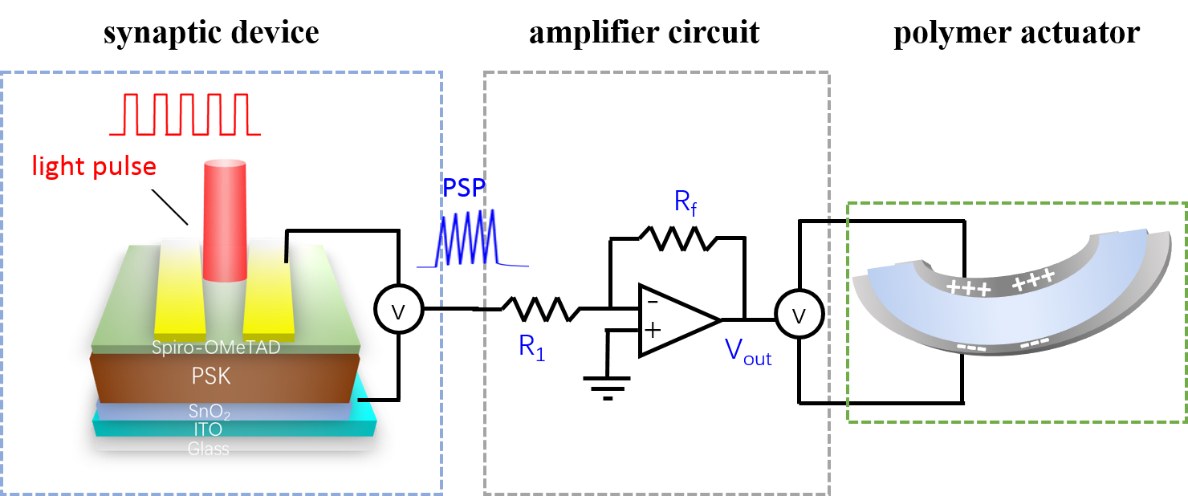


Fig. S5. Construction of synaptic device-amplifier circuit-polymer actuator system.

**IV. Self-powered synaptic characteristics of optoelectronic perovskite device. (Fig. S6-S8)**

In neuroscience, an excitatory postsynaptic potential (EPSP) is a postsynaptic potential that increases the likelihood that the postsynaptic neuron will fire an action potential. This temporary depolarization of postsynaptic membrane potential, caused by the flow of positively charged ions into the postsynaptic cell, is a result of opening ligand-gated ion channels. These are the opposite of inhibitory postsynaptic potentials (IPSPs), which usually result from the flow of negative ions into the cell or positive ions out of the cell. EPSPs can also result from a decrease in outgoing positive charges, whereas IPSPs are sometimes caused by an increase in positive charge outflow. The flow of ions that causes an EPSP is an excitatory postsynaptic current (EPSC).


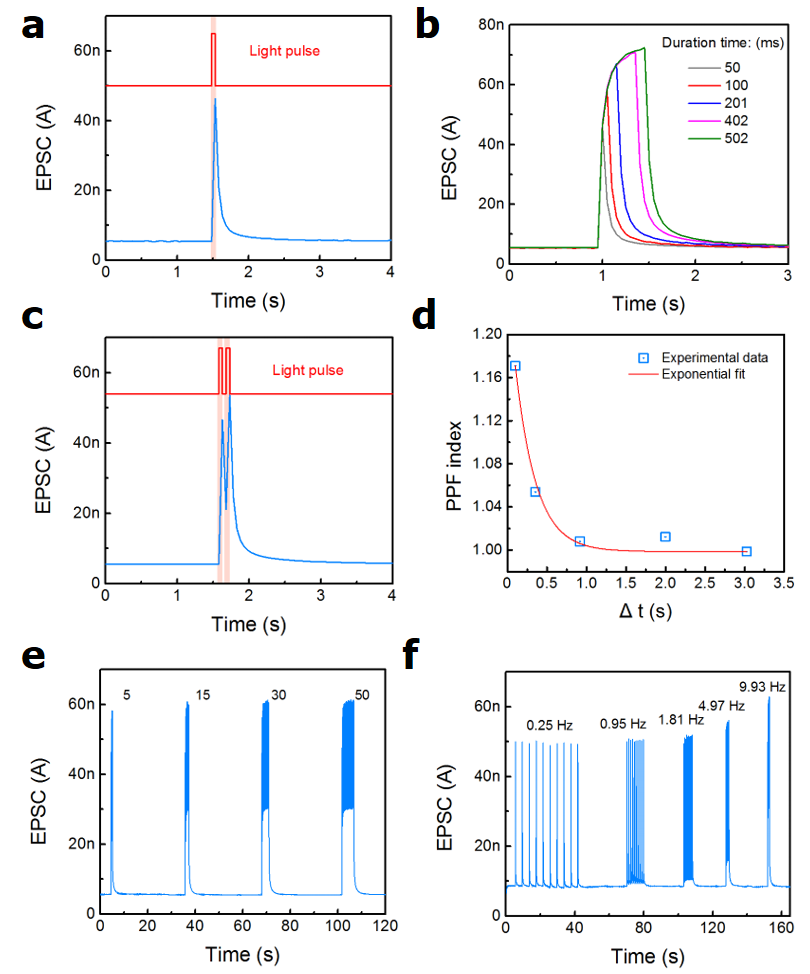


Fig. S6. Self-powered synaptic characteristics of optoelectronic perovskite device. (a) Transient EPSC of perovskite synaptic device in response to one optical spike. (b)  EPSC vs. spike duration. (c) Change in EPSC in response to a pair of presynaptic optical pulses. *A*_1_ and *A*_2_ represent change in PSC at first and second spike, respectively. (d) Paired-pulse facilitation (PPF) index vs. interval between pairs of excitatory stimuli. (e) EPSC vs. number of optical spikes. (f) Spike-frequency-dependent EPSC amplitude triggered by a train of 10 light spikes.


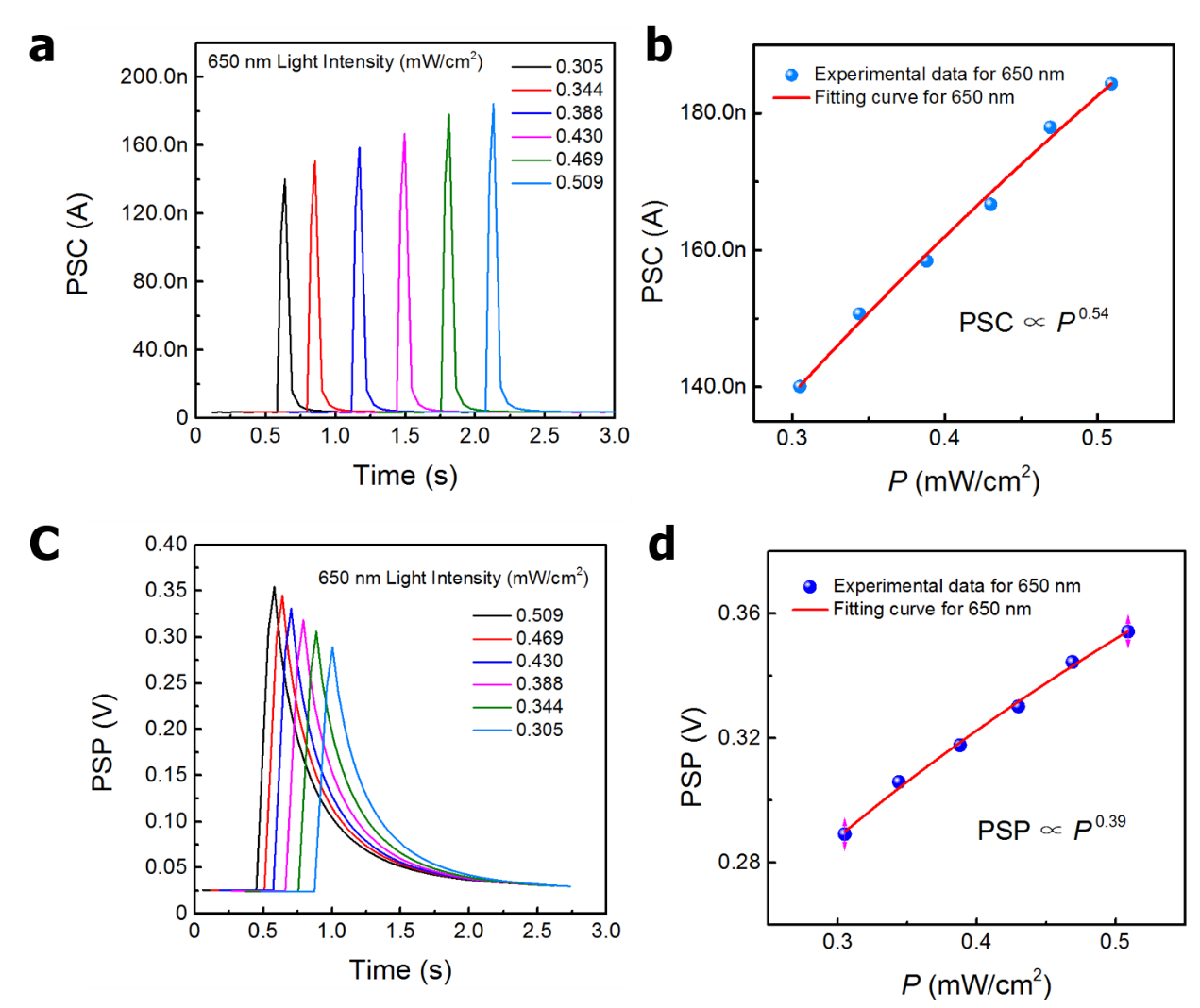


Fig. S7. The synaptic characteristics of optoelectronic perovskite device measured at different incident light intensities under illumination using light of wavelength 650 nm. (a) PSC and (c) PSP values vs. intensity optical spikes. Postsynaptic current (b)/potential (d) vs. light irradiation power.


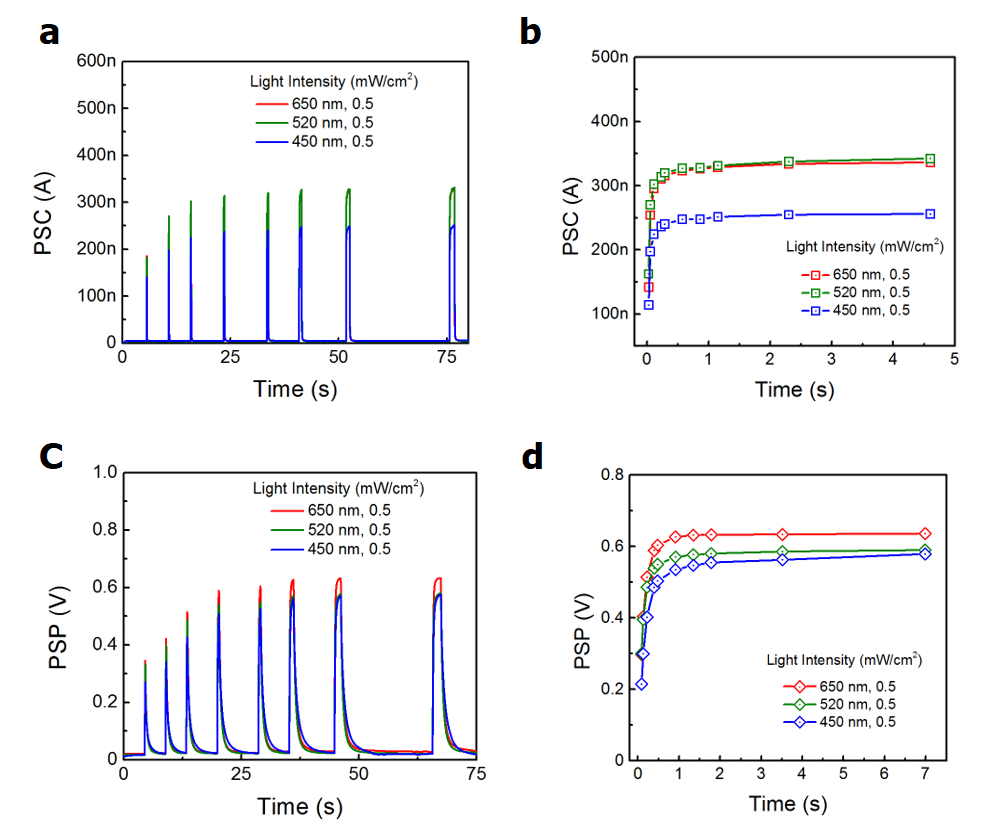


Fig. S8. The synaptic characteristics of optoelectronic perovskite device vs. incident light wavelengths at 0.5 mW/cm^2^ incident light intensities. The (a) PSC and (c) PSP during 0.5‑mW/cm^2^ intensity optical spikes with wavelengths of 450 nm, 520 nm or 650 nm. (b) PSC and (d) PSP values vs. duration at various incident light wavelengths.


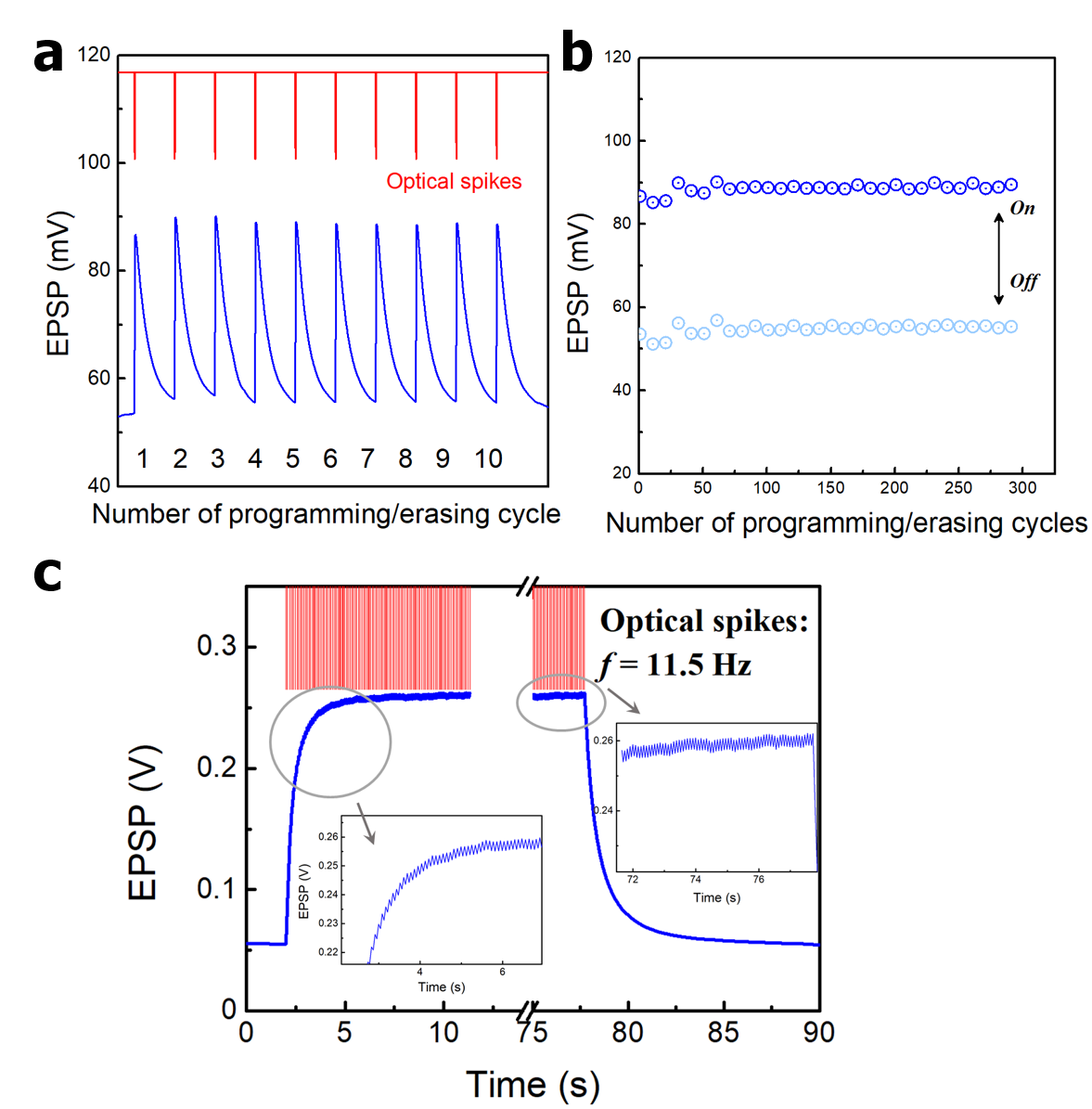


**Fig. S9.** (a) EPSP vs. number of optical spikes with *f* = 0.3 Hz. (b) Variation of the off- and on-PSP from the initial to 300 of programming/erasing cycles with an interval of 10 cycles in the endurance performance test. The device is programmed by the on-light pulse (*f* = 0.3 Hz), erased by the off-light pulse, exhibiting the capability of cyclic endurance. (c) EPSP vs. number of optical spikes with *f* = 11.5 Hz.

Table S1. Summary of typical artificial nervous system.

| **Typical relative work** | **Sensory stimuli** | **Communicate** | | **Processing** | | **Actuation** |
| --- | --- | --- | --- | --- | --- | --- |
|  |  | **Neural coding** | **Signal transmission** | **Processing unit** | **Self-powered** |  |
| Organic artificial afferent nerve [7] | Pressure | Rate coding | Voltage spikes | Synaptic transistor | - | A detached cockroach leg |
| Organic optoelectronic sensorimotor synapse [10] | Light | Amplitude | DC | Synaptic transistor | - | Electrochemical actuator |
| Artificial peripheral nervous system [44] | Pressure & temperature | Rate coding | Electrical voltage pulse codes | Microcontrollers | - | - |
| Optoelectronic spiking afferent nerve [13] | Pressure | Rate coding | Optical spikes | Synaptic photomemristor | - | - |
| NeuTap neuron [45] | Pressure | Amplitude | DC | Synaptic transistor | - | - |
| Artificial haptic neuron system [46] | Pressure | Amplitude | DC | Nafion memristor |  | - |
| Bimodal artificial sensory neuron (BASE) [21] | Pressure & light | Amplitude | DC | Synaptic transistor | - | Cultured skeletal myotubes |
| Contact-electrification-activated artificial afferents [47] | Pressure | Amplitude | DC | Synaptic transistor | - | - |
| mechano-photonic artificial synapse [23] | Pressure & light | Amplitude | DC | Synaptic transistor | - | - |
| Artificial Multisensory Integration Nervous System with Haptic and Iconic Perception Behaviors [48] | Pressure & light | Amplitude | DC | Synaptic transistor | - | - |
| This work | Pressure & light | Rate coding | Optical spikes | Photoelectric synapse | √ | Electrochemical actuator |
